# *d*-Dimensional Classical Heisenberg Model with Arbitrarily-Ranged Interactions: Lyapunov Exponents and Distributions of Momenta and Energies

**DOI:** 10.3390/e21010031

**Published:** 2019-01-04

**Authors:** Antonio Rodríguez, Fernando D. Nobre, Constantino Tsallis

**Affiliations:** 1 GISC, Departamento de Matemática Aplicada a la Ingeniería Aeroespacial, Universidad Politécnica de Madrid, Plaza Cardenal Cisneros s/n, 28040 Madrid, Spain; 2 Department of Physics, University of Warwick, Coventry CV4 7AL, UK; 3 Centro Brasileiro de Pesquisas Físicas and National Institute of Science and Technology for Complex Systems, Rua Dr. Xavier Sigaud 150, Rio de Janeiro 22290-180, Brazil; 4 Santa Fe Institute, 1399 Hyde Park Road, Santa Fe, NM 87501, USA; 5 Complexity Science Hub Vienna, Josefstädter Strasse 39, 1080 Vienna, Austria

**Keywords:** complex Hamiltonian systems, nonextensive statistical mechanics, long-ranged-interacting thermostatistics, lyapunov exponents

## Abstract

We numerically study the first-principle dynamics and thermostatistics of a *d*-dimensional classical inertial Heisenberg ferromagnetic model (d=1,2,3) with interactions decaying with the distance $r_{ij}$ as $1/r_{ij}^{\alpha }$ (α≥0), where the limit α=0 (α→∞) corresponds to infinite-range (nearest-neighbour) interactions, and the ratio α/d>1 (0≤α/d≤1) characterizes the short-ranged (long-ranged) regime. By means of first-principle molecular dynamics we study: (i) The scaling with the system size N of the maximum Lyapunov exponent λ in the form $\lambda ∼N^{-\kappa }$, where κ(α/d) depends only on the ratio α/d; (ii) The time-averaged single-particle angular momenta probability distributions for a typical case in the long-range regime 0≤α/d≤1 (which turns out to be well fitted by *q*-Gaussians), and (iii) The time-averaged single-particle energies probability distributions for a typical case in the long-range regime 0≤α/d≤1 (which turns out to be well fitted by *q*-exponentials). Through the Lyapunov exponents we observe an intriguing, and possibly size-dependent, persistence of the non-Boltzmannian behavior even in the α/d>1 regime. The universality that we observe for the probability distributions with regard to the ratio α/d makes this model similar to the α-XY and α-Fermi-Pasta-Ulam Hamiltonian models as well as to asymptotically scale-invariant growing networks.

## 1. Introduction

Although nature is strictly quantum at the microscopic level, classical systems of particles have been studied for decades, usually considered as good approximations in some limiting cases. In what concerns magnetism, classical spin models have succeeded in providing good qualitative agreement with experiments on several materials [1,2,3]. Many of these investigations focused on equilibrium properties of models characterized by nearest-neighbour interactions (see, e.g., Refs. [4,5,6,7,8,9,10,11]). In contrast, a paradigmatic example dealing with infinite-range interactions, the so-called Hamiltonian Mean Field (HMF) model, consisting of *N* fully coupled classical XY rotators, was introduced in [12]. This was done through the addition of a kinetic term in the Hamiltonian of a classical XY model, so that equations of motion could be derived, leading to the possibility of investigating its dynamic properties by a direct integration of these equations. Since then, a vast amount of papers devoted to the study of the HMF model have put forward its anomalous properties [13,14,15,16,17,18,19,20,21,22,23]: existence of negative specific heat, quasistationary states (QSSs), nonmaxwellian velocity probability distributions, anomalous diffusion and ergodicity breaking, among others.

The HMF model was further generalized into the so-called α-XY model [24], via the introduction of an interaction term decaying with the distance *r* between rotators as $r^{-\alpha }$. The parameter α≥0 controls the range of the interaction, with the limits α=0 and α→∞ corresponding respectively to the HMF and nearest-neighbour models. For the α-XY model defined on *d*-dimensional lattices, the mean potential energy per particle diverges in the thermodynamic limit for 0≤α/d≤1. Consequently, serious mathematical difficulties emerge in the thermodynamic limit for the Boltzmann-Gibbs (BG) partition function. Nowadays, it has become evident that BG statistical mechanics fails in describing appropriately systems generically characterized by relevant long-range correlations (e.g., due to long-range interactions). In such cases, the classification of thermodynamical quantities as either extensive or intensive is not sufficient, since one frequently finds a third category, the one of nonextensive quantities.

These kinds of difficulties have led to generalizations of BG statistical mechanics, and the most widely used theory for dealing with such systems so far is nonextensive statistical mechanics [25]. This theory emerged from the proposal of a generalized entropy, known as $S_{q}$, characterized by a real index *q* [26],

(1) $$S_{q}=k\sum_{i=1}^{W}p_{i}\left(\ln_{q}\frac{1}{p_{i}}\right)\;,$$

where we have introduced the *q*-logarithm definition,

(2) $$\ln_{q}u=\frac{u^{1-q}-1}{1-q}\;;\;\left(\ln_{1}u=\ln u\right)\;.$$

Therefore, one recovers BG entropy as $\lim_{q\to 1}S_{q}=S_{\mathrm{BG}}$, whereas in the microcanonical ensemble, where all microstates present equal probability, $p_{i}=1/W$, Equation (1) becomes,

(3) $$S_{q}=k\ln_{q}W\;.$$

Since then, a considerable progress was achieved, leading to generalized functions, distributions, and important equations of physics. In particular, the *q*-Gaussian distribution, which generalizes the standard Gaussian, appears naturally by extremizing the entropy $S_{q}$ [25],

(4) $$P_{q}\left(u\right)=P_{0}\exp_{q}\left(-\beta u^{2}\right)\;,$$

with

(5) $$\exp_{q}\left(u\right)={\left[1+\left(1-q\right)u\right]}_{+}^{1/(1-q)}\;;\;\left(\exp_{1}\left(u\right)=\exp\left(u\right)\right)\;,$$

where $P_{0}\equiv P_{q}\left(0\right)$ and ${\left[y\right]}_{+}=y$, for y>0 (zero otherwise). Above, the *q*-exponential function appears precisely as the inverse function of the *q*-logarithm of Equation (2), i.e., $\exp_{q}\left(\ln_{q}u\right)=\ln_{q}\left(\exp_{q}\left(u\right)\right)=u$.

The α-XY model has also been largely studied [27,28,29,30,31,32,33,34] and, in the *d*-dimensional case, it was shown [29] that in its high-energy regime, the maximum Lyapunov exponent scales with the number $N\equiv N^{d}$ of rotators as $\lambda ∼N^{-\kappa }$, where the exponent κ(α,d) depends on α and *d* through the ratio α/d, as in the case of other quantities, with κ>0 in the long-range regime (α/d≤1) and κ=0 (thus yielding positive Lyapunov exponents) in the short-range regime (α/d>1). In addition, *q*-Gaussians have been obtained for the time-averaged momenta probability distributions not only before, but even after the crossover to the very-large-time QSS, with a value of the entropic index $q\equiv q_{p}\left(\alpha /d\right)$ depending again on the ratio α/d [34]. The usual Maxwellian distributions are recovered as α/d increases above unity. A curious behavior occurs also for the ensemble-averaged momenta probability distributions. The same departure from the BG predictions is observed for the time-averaged energy distributions; in this case, instead of the usual BG exponential, *q*-exponential probability distributions are obtained, with $q\equiv q_{E}\left(\alpha /d\right)$ [34]. Similar universality features have been recently obtained in quite different contexts, such as a generalized Fermi-Pasta-Ulam model with long-range interactions [35,36,37,38,39], complex networks with preferential attachment growth [40,41], and a system of particles under overdamped motion interacting repulsively with power-law interactions [42].

By considering three-dimensional rotators (instead of planar ones), the corresponding classical inertial ferromagnetic Heisenberg model is obtained; this has not been very much explored in the literature [43,44,45,46,47]. In the fully coupled (infinite-range interactions) version, QSSs have also been observed, implying that, just as in the HMF model, the thermodynamic and time limits do not commute: if the N→∞ limit is taken first, the system will remain in the QSS, whereas if the t→∞ limit is considered first, the system may attain the Boltzmann temperature after a transient time, which was shown to increase with the system size [44,45,46,47]. Inspired by the α-XY model, the classical inertial ferromagnetic Heisenberg model was also generalized by introducing an interaction term decaying with the distance *r* between rotators as $r^{-\alpha }$ (α≥0) [47]. Such an α-Heisenberg model, studied up to the moment only in dimension d=1, exhibited a clear QSS in the long-range-interaction regime, with its duration decreasing for increasing values of α. In this paper, we extend the α-Heisenberg model to dimensions d=2 and 3, and show that the scaling with the system size of the maximum Lyapunov exponent follows the same trend as in the above indicated α-XY model. In addition, we calculate numerically the probability distributions for the time-averaged momenta, as well as the energy distributions, in order to get α/d-dependent entropic indexes. The anomalous (non-Maxwellian for the momenta and nonexponential for the energies) distributions persist well beyond the α=d threshold and intriguingly coexist with positive values of the maximal Lyapunov exponent, at least for the sizes that have been numerically attained.

## 2. The Model

The α-Heisenberg inertial model consists of a collection of N interacting rotators, represented by three-component rotating vectors ${\vec{S}}_{i}$ with unit norm, located at fixed positions on a *d*-dimensional hypercubic lattice of linear size *N* ($N\equiv N^{d}$).The Hamiltonian is given by

(6) $$H=K+V_{\alpha }=\frac{1}{2}\sum_{i=1}^{N}{\vec{L}}_{i}^{2}+\frac{1}{2\tilde{N}}\sum_{i=1}^{N}\sum_{\begin{array}{c} j=1\\ j\neq i \end{array}}^{N}\frac{1-{\vec{S}}_{i}\cdot {\vec{S}}_{j}}{r_{ij}^{\alpha }},$$

where α≥0 controls the interaction range, $r_{ij}$ denotes the distance between rotators *i* and *j*, the angular momenta ${\vec{L}}_{i}$ coincide with the angular velocities since we are considering unit moments of inertia. Moreover, the prefactor with $\tilde{N}\left(\alpha ,d\right)\equiv \sum_{j\neq i}\frac{1}{r_{ij}^{\alpha }}$ in the potential energy is convenient in order to get a total potential energy $V_{\alpha }$ which scales linearly with N [25], like the total kinetic energy.

### 2.1. The α=0 and α→∞ Limiting Cases

It is easily seen that, for α=0, $\tilde{N}\left(0,d\right)=N-1∼N$, so in the infinite-range case the total potential energy reduces to

(7) $$V_{0}=\frac{1}{2N}\sum_{i=1}^{N}\sum_{j=1}^{N}\left(1-{\vec{S}}_{i}\cdot {\vec{S}}_{j}\right)=\frac{N}{2}-\frac{1}{2N}\sum_{i=1}^{N}{\vec{S}}_{i}\sum_{j=1}^{N}{\vec{S}}_{j}=\frac{N}{2}\left(1-M^{2}\right),$$

where the restriction j≠i has been trivially dismissed since ${\vec{S}}_{i}^{\;2}=1$, and the order parameter *M* is the norm of the total magnetization vector

(8) $$\vec{M}=\frac{1}{N}\sum_{i=1}^{N}{\vec{S}}_{i}.$$

In addition, following the usual prescription for the kinetic temperature, $T=\frac{2}{nN}\langle K\rangle =\frac{1}{N}\langle K\rangle$, where n=2 represents the number of degrees of freedom per spin (three components of the spin vector minus the constant norm restriction). Thus, considering the energy per particle $U\equiv \frac{\langle H\rangle }{N}$ in Hamiltonian (6) with α=0 together with Equation (7), one gets

(9) $$U=T+\frac{1}{2}\left(1-M^{2}\right),$$

which, together with the consistency equation for the magnetization [1,2,3],

(10) M=L(M/T),

with the Langevin function $L\left(x\right)\equiv \coth x-\frac{1}{x}$, allows us to determine a relation between the thermodynamical variables of the model, which undergoes a second-order phase transition for the critical values $\left(T_{c},U_{c}\right)=\left(\frac{1}{3},\frac{5}{6}\right)$. The caloric curve, as well as the M−T curve and some other details of the model can be found in Ref. [47]. It can be shown that [27], within BG statistical mechanics, Equations (9) and (10) are not only valid for α=0 but also throughout the long-range regime 0≤α/d<1.

No analytical results are available in the short-range regime except for the limiting case α→∞, which corresponds to nearest-neighbors interactions, where $\tilde{N}\left(\infty ,d\right)=2d$ (that is, the number of nearest-neighbour spins on a *d*-dimensional hypercubic lattice) and the potential energy reduces to

(11) $$V_{\infty }=\frac{1}{2d}\sum_{\langle i,j\rangle }\left(1-{\vec{S}}_{i}\cdot {\vec{S}}_{j}\right)\;,$$

where 〈i,j〉 indicates that sites labelled *i* and *j* are nearest neighbours. The equation for the internal energy in the one-dimensional case reads

(12) $$U=T+\frac{1}{2}\left(1-L\left(1/2T\right)\right),$$

while M=0,∀T≠0 (there is no phase transition in the d=1 short-range regime).

### 2.2. Equations of Motion

From the definition ${\vec{L}}_{i}={\vec{S}}_{i}\times {\dot{\vec{S}}}_{i}$ one trivially gets ${\dot{\vec{S}}}_{i}={\vec{L}}_{i}\times {\vec{S}}_{i}$. In addition, ${\dot{\vec{L}}}_{i}={\vec{S}}_{i}\times {\ddot{\vec{S}}}_{i}=-{\vec{S}}_{i}\times \frac{\partial V_{\alpha }}{\partial {\vec{S}}_{i}}$, so the equations of motion are given by

(13) $$\begin{array}{cc} {\dot{\vec{S}}}_{i} & ={\vec{L}}_{i}\times {\vec{S}}_{i} \end{array}$$

(14) $$\begin{array}{cc} {\dot{\vec{L}}}_{i} & ={\vec{S}}_{i}\times {\vec{M}}_{i} \end{array}$$

where

(15) $${\vec{M}}_{i}\equiv -\frac{\partial V_{\alpha }}{\partial {\vec{S}}_{i}}=\frac{1}{\tilde{N}}\sum_{j\neq i}^{N}\frac{{\vec{S}}_{j}}{r_{ij}^{\alpha }},$$

carries the contribution of the interactions of spin *i* with all (N−1) other spins. This quantity becomes the total magnetization vector of (8) when α=0, and the sum $\frac{1}{2d}\sum_{\langle i,j\rangle }{\vec{S}}_{j}$, extended to the nearest neighbours of spin *i*, when α=∞. From Equations (13) and (14) not only follows the aforementioned conservation of the spin norms, since ${\dot{\vec{S}}}_{i}^{\;2}=2{\vec{S}}_{i}\cdot {\vec{S}}_{i}=2{\vec{S}}_{i}\cdot \left({\vec{L}}_{i}\times {\vec{S}}_{i}\right)=0$, but also the conservation of the total angular momentum $\vec{L}=\sum_{i=1}^{N}{\vec{L}}_{i}$, since $\dot{\vec{L}}=\sum_{i=1}^{N}{\dot{\vec{L}}}_{i}=\frac{1}{\tilde{N}}\sum_{i=1}^{N}\sum_{j\neq i}\frac{{\vec{S}}_{i}\times {\vec{S}}_{j}}{r_{ij}^{\alpha }}=\vec{0}$.

## 3. Results

We have numerically integrated the equations of motion (13) and (14) by means of the standard fourth-order Runge-Kutta algorithm with an integration step h=0.02. It is well known that numerical methods, like the one used herein, present an energy drift for long-time simulations [48,49,50]; using the integration step above, we have checked that the total energy was preserved within a relative precision $10^{-5}$.We have considered two different kinds of initial conditions. In the first one we take random sets of angular momenta $\left\{{\vec{L}}_{i}\right\}$, with components drawn from a symmetric uniform distribution and then redefined in order to yield zero total momentum. As for the spins $\left\{{\vec{S}}_{i}\right\}$, two of their components are randomly chosen, while the third one is obtained by the orthogonality restriction ${\vec{S}}_{i}\cdot {\vec{L}}_{i}=0$(∀i). Then, we get spins of unit length dividing by the norm. Finally, the angular momenta are rescaled so as to obtain the desired total energy per particle *U* which remains virtually constant throughout our microcanonical ensemble simulations. This process yields a poorly magnetized initial state with $\vec{M}\left(0\right)∼\vec{0}$ and $V_{\alpha }\left(0\right)∼0.5$. In the second kind of initial conditions, we take all the spins equal and normalized, so as to get a fully magnetized state with M(0)=1 and zero potential energy. Then we randomly choose the angular momenta in the perpendicular plane to finally translate and rescale them to get zero total angular momentum and the desired total energy. Large time-limit qualitative and quantitative results coincide with both kinds of initial conditions.

### 3.1. Time-Averaged Momenta and Energy One-Particle Distributions

**Figure 1:**
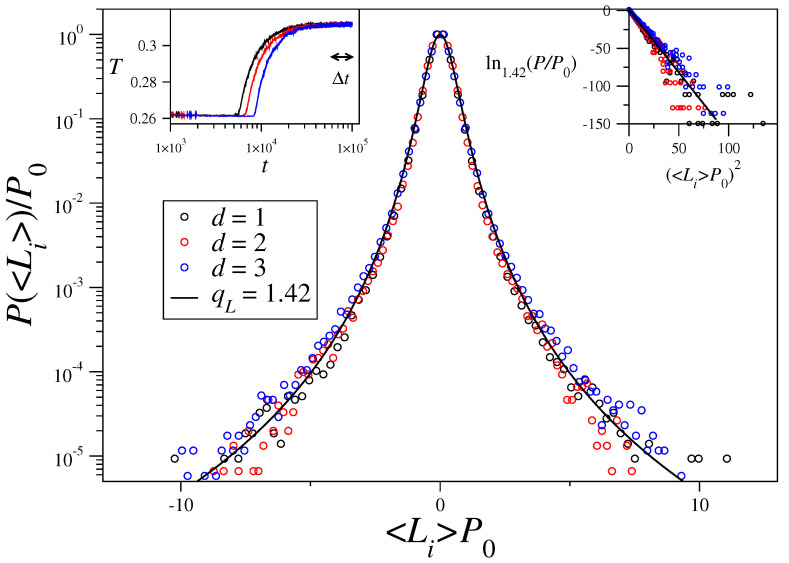
Probability distribution functions of time-averaged angular momenta $P(\langle L_{i}\rangle )$ are represented in conveniently rescaled variables, for one-, two-, and three-dimensional systems with N= 262,144 rotators and α/d=0.9. The three data sets collapse into a *q*-Gaussian (full line), $P\left(\langle L_{i}\rangle \right)=P_{0}\exp_{q_{L}}\left(-\beta_{L}{\left(\langle L_{i}\rangle P_{0}\right)}^{2}\right)$ (cf. Equation (4)), with $q_{L}\left(\alpha /d\right)=1.42$ and $\beta_{L}=4.44$; the maximum values $P_{0}\equiv P\left(\langle L_{i}\rangle =0\right)$ varied slightly with the lattice dimensionality (see text). In the left inset we preset the evolution in time of the kinetic temperature. A first plateau at a temperature close to $T_{\mathrm{QSS}}\left(\alpha /d\right)=U-\frac{1}{2}$, whose duration depends on *d*, is observed. After the transition to the second plateau, the temperature reaches the equilibrium value $T_{\mathrm{BG}}\left(\alpha /d\right)=0.3118$ predicted by the BG caloric curve [33]; the time window [60,000, 100,000] used for the time averages is indicated. In the inset on the right we represent the same data in the *q*-logarithm $\left(q_{L}=1.42\right)$ of Equation (2) versus ${\left(\langle L_{i}\rangle P_{0}\right)}^{2}$, where the slope of the full straight line yields the value of $\beta_{L}$.

As already shown in Ref. [47], for values of the total energy below the critical one but close enough to the transition, after a short initial transient, the kinetic temperature of the system, $T\left(t\right)=\frac{1}{N}\langle K\left(t\right)\rangle$, decreases to a first plateau value given by T=U−1/2 (consider M=0 in Equation (9)), corresponding to a nonmagnetized state. Only after waiting long enough, the system abandons the QSS when the temperature as well as the magnetization rise to reach the values predicted by Equation (9) and stays forever in this second plateau, which is non Boltzmannian though its temperature coincides with that associated with BG statistics. It is in this (nearly) final QSS where we choose a time window $\left[t_{0},t_{f}\right]$ of width Δt=W×h and calculate the averaged-in-time momenta as $\langle {\vec{L}}_{i}\rangle =\frac{1}{W+1}\sum_{k=0}^{W}{\vec{L}}_{i}\left(t_{0}+kh\right)$. Then we put together all the 3N averaged-in-time angular momenta components to build the corresponding histograms. In Figure 1 we present the probability distribution functions of time-averaged angular momenta $P(\langle L_{i}\rangle )$ for one-, two-, and three-dimensional systems. These distributions were computed for systems with $\vec{M}\left(0\right)∼0$ initial conditions, total energy per particle $U=0.76<U_{c}=5/6$, size $N=262144={\left(512\right)}^{2}={\left(64\right)}^{3}$ (corresponding to d=1,2, and 3), and α/d=0.9, that is, in the long-range-interaction regime. A clear collapse of all three distributions into a *q*-Gaussian (cf. Equation (4)) is exhibited, with $q_{L}\left(\alpha /d\right)=1.42$ and $\beta_{L}=4.44$; the maximum values $P_{0}\equiv P\left(\langle L_{i}\rangle =0\right)$ fluctuated slightly with the lattice dimensionality, being found as $P_{0}=0.27\;\left(d=1\right)$, $P_{0}=0.19\;\left(d=2\right)$, and $P_{0}=0.22\;\left(d=3\right)$.Further results exhibiting values of $q_{L}$ as a function of the ratio α/d will be published elsewhere.

**Figure 2:**
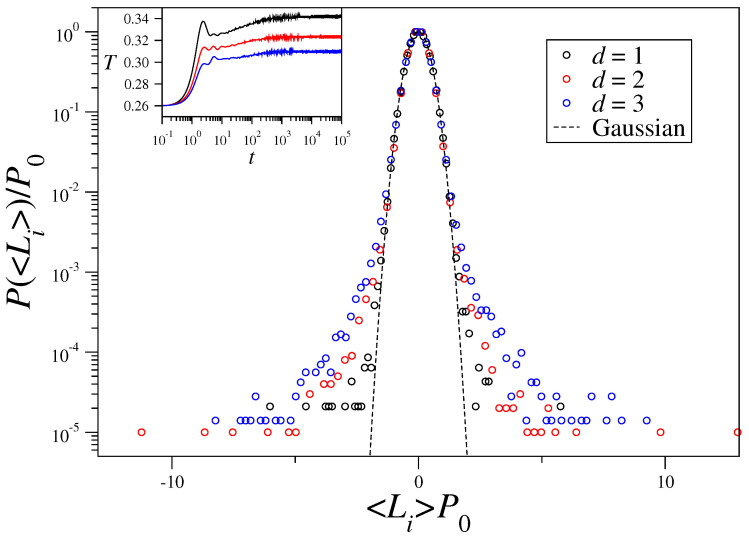
Probability distribution functions of time-averaged angular momenta $P(\langle L_{i}\rangle )$ are represented in conveniently rescaled variables $\left(P_{0}\equiv P\left(\langle L_{i}\rangle =0\right)\right)$ for one-, two-, and three-dimensional systems with N= 262,144 rotators and α/d=1.6. The data cannot be adequately fitted by a single *q*-Gaussian; a Gaussian (dashed line) is exhibited for comparison, showing a good fitting around the central region only, i.e., for small values of $\langle L_{i}\rangle$. The left inset exhibits the time evolution of the kinetic temperature: one sees that the QSS is not present in the short-range regime and the system rapidly reaches its equilibrium temperature which depends on α and *d* separately; the same time window shown in Figure 1 has been used for the time averages.

In Figure 2 we exhibit the probability distribution functions of time-averaged angular momenta $P(\langle L_{i}\rangle )$ for α/d=1.6, that is, in the short-range regime, with the same initial conditions and parameters used in Figure 1. Now, the collapse into a single curve occurs only around the origin; next we point out possible reasons for the vital differences between the probability distributions shown in Figure 1 and Figure 2. (i) The distinction in the time evolution of the kinetic temperature, shown in the left insets in both figures. Particularly, one should emphasize that the equilibrium temperature (along which time averages $\langle L_{i}\rangle$ were computed) in Figure 2 depends on α and *d* separately. (ii) Finite-size effects, which become more significant in the limit of short-range interactions, might be playing an important role. (iii) At the BG equilibrium, the corresponding short-range-interaction models present further relevant distinctions, e.g., in d=1 there is no phase transition, whereas an ordered state exists in d=3 [1,2,3]. (iv) The maximum Lyapunov exponents show significant differences in the cases investigated in Figure 1 and Figure 2, as we shall see below.

**Figure 3:**
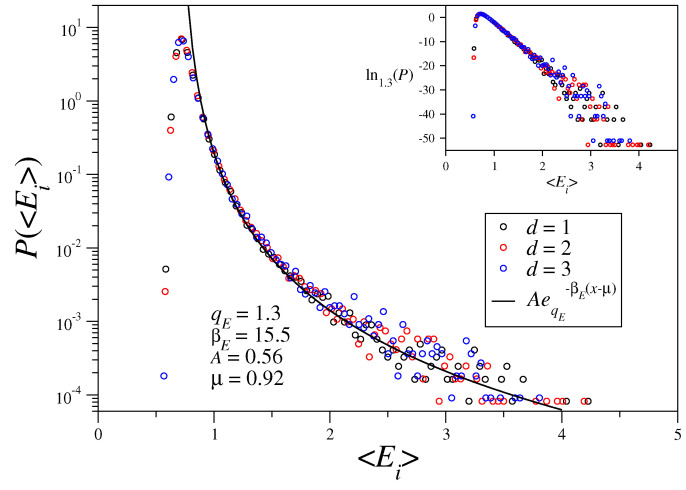
Probability distribution functions of time-averaged individual energies $P(\langle E_{i}\rangle )$ are represented versus $\langle E_{i}\rangle$ for one-, two-, and three-dimensional systems. The parameters are the same as in Figure 1, i.e., N= 262,144 rotators, α/d=0.9, and the same time window. All data are well fitted by a shifted *q*-exponential (cf. Equation (5)) of the form $Ae_{q_{E}}^{-\beta_{E}\left(\langle E_{i}\rangle -\mu \right)}$, with the values of *A*, $q_{E}$, $\beta_{E}$, and μ specified. In the inset we exhibit the same data in the *q*-logarithm $\left(q_{E}=1.3\right)$ of Equation (2) versus $\langle E_{i}\rangle$, which, as expected, approaches a straight line.

We have also calculated the probability distributions of time-averaged individual energies, i.e., $\langle E_{i}\rangle =\frac{1}{W+1}\sum_{k=0}^{W}E_{i}\left(t_{0}+kh\right)$, with $E_{i}=\frac{1}{2}{\vec{L}}_{i}^{2}+\frac{1}{2\tilde{N}}\sum_{j\neq i}^{N}\frac{1-{\vec{S}}_{i}\cdot {\vec{S}}_{j}}{r_{ij}^{\alpha }}$. Results for the long-range-interaction regime, with α/d=0.9 and the same initial conditions and parameter values considered in Figure 1, are shown in Figure 3. Again, all data collapse, now in the energy range $E_{i}\geq \mu$ (see details in Figure 3); since different dimensions present distinct densities of states, the data does not collapse near the origin. One should call attention to the fact that the value $q_{E}=1.3$ of Figure 3 coincides with the recent result for the α-XY model, obtained also with α/d=0.9 [34]. Moreover, as found in the study of the α-XY model [34], a curious discrepancy in the values of the index *q* occurred in the estimates of Figure 1 and Figure 3, i.e., $q_{L}>q_{E}$. Whether this may be a real effect, or a consequence of finite-size effects, remains a point for further investigation. Moreover, similarly to Figure 2, the corresponding probability distributions $P(\langle E_{i}\rangle )$ in the short-range regime (for α/d=1.6), do not reveal a collapse of the curves into a *q*-exponential, so that the significant distinctions in the short-range regime persist.

### 3.2. Nearest-Neighbour Limit

**Figure 4:**
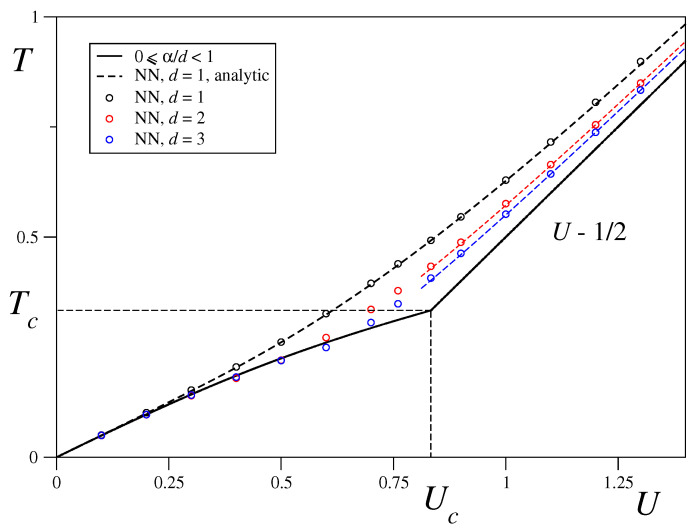
Analytic caloric curve for the Boltzmann-Gibbs (BG) long-range (full line) and one-dimensional nearest-neighbour (black dashed line) models compared with nearest-neighbour (NN) molecular dynamics simulation results for dimensions d=1, 2 and 3 and system size N= 46,656 $={\left(216\right)}^{2}={\left(36\right)}^{3}$. Fully magnetized initial state has been considered in all cases. The dashed lines connecting d=2 and d=3 numerical results for $U>U_{c}$ correspond to Equation (12) with L evaluated at 1/2dT.

In order to shed more light on the short-range regime, we shall focus now on the limiting α→∞ case. We have calculated the equilibrium temperature, as well as the magnetization as a function of the total energy per particle and the dimension. Analytical results are available only for the caloric curve in the one-dimensional case, as given by Equation (12) and shown in Figure 4, where we obtain an excellent agreement between the theoretical results and the numerical ones obtained in our microcanonical ensemble simulations with M(0)=1 initial conditions (which are mandatory in this case since, for small values of the constant total energy per particle we need to have vanishing initial potential energy and the potential energy for the $\vec{M}\left(0\right)∼0$ initial conditions is V∼0.5) and N= 46,656. The caloric curve corresponding to the long-range regime 0≤α/d<1 is also shown in Figure 4 where it can be seen that the temperature coincides in both limits α→0 and α→∞ in all dimensions within the energy range 0≤U≤0.25. For larger values of *U*, the equilibrium temperature decreases with the dimension in the nearest neighbours limit, which is to be expected since the number of connections and thus the potential energy increases with the dimension while the total energy is kept constant so the kinetic energy and thus the temperature must decrease. Finally, it is worth mentioning that in the energy range $U>U_{c}$, the temperature versus energy curves for d>1 are surprisingly well approximated by Equation (12) with the Langenvin function evaluated at 1/2dT.

**Figure 5:**
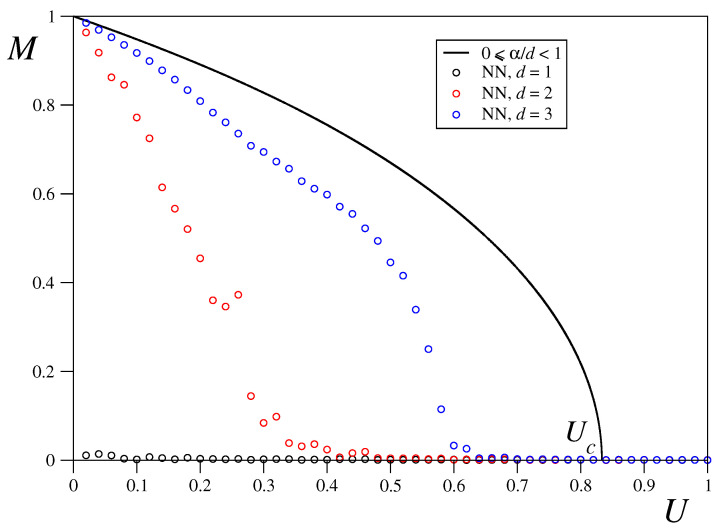
Analytic M−U curve for the BG long-range (full line) model compared with nearest-neighbour (NN) molecular dynamics simulation results for dimensions d=1, 2 and 3 and system size N= 46,656 $={\left(216\right)}^{2}={\left(36\right)}^{3}$. Fully magnetized initial state has been considered in all cases.

Figure 5 shows the *M* vs. *U* curve for the long-range regime compared with the nearest neighbours regime curve for 1, 2 and 3 dimensions with the same parameter values and initial conditions used in Figure 4. As expected, in one dimension one gets M=0 for any value of *U* since there is no phase transition for d=1. For d=3 the order parameter *M* vanishes at the critical value $U\equiv U_{3d}∼0.6$ for which the ferromagnetic-paramagnetic transition takes place. In dimension d=2, it is observed a less sharp decay of the magnetization, which vanishes for $U\equiv U_{2d}∼3.5$.

### 3.3. Size-Scaling of the Largest Lyapunov Exponent

**Figure 6:**
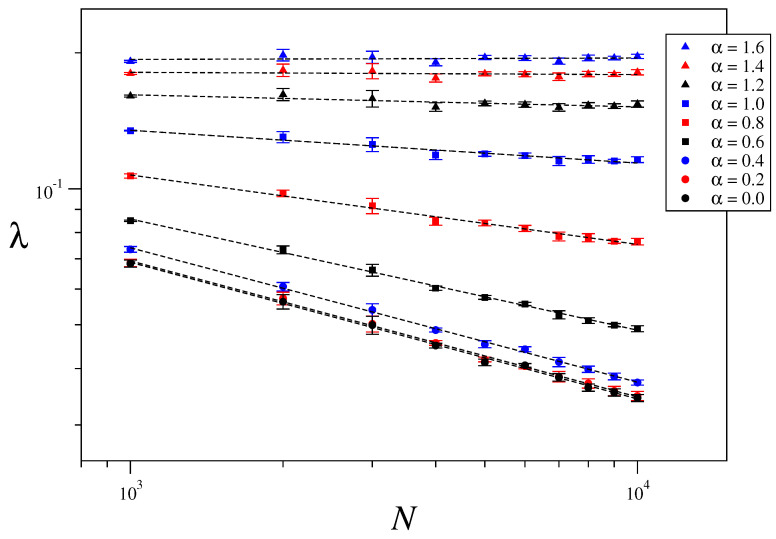
Maximum Lyapunov exponent as a function of the system size for U=5.0, d=1 and different values of α. The considered system sizes are $N=10^{3},2\times 10^{3},3\times 10^{3}\cdots ,9\times 10^{3}$ and $10^{4}$.

**Figure 7:**
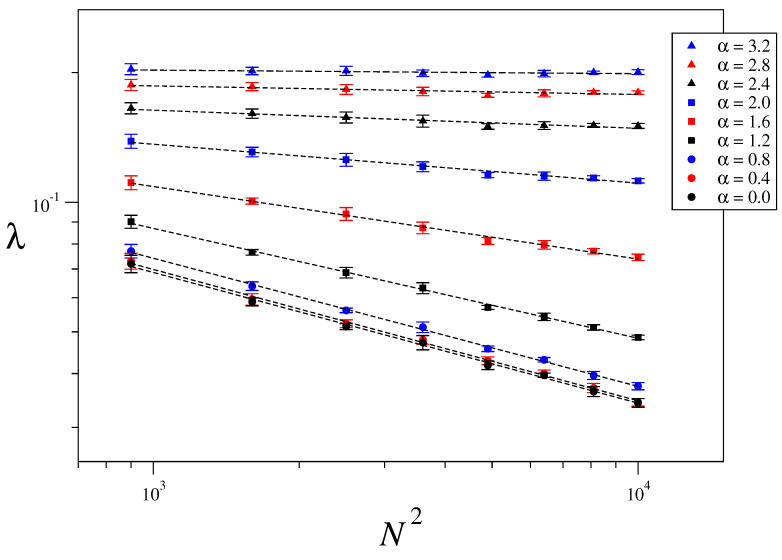
Maximum Lyapunov exponent as a function of the system size for U=5.0, d=2 and different values of α. The considered system sizes are $N={\left(30\right)}^{2},{\left(40\right)}^{2},{\left(50\right)}^{2}\cdots ,{\left(100\right)}^{2}$.

**Figure 8:**
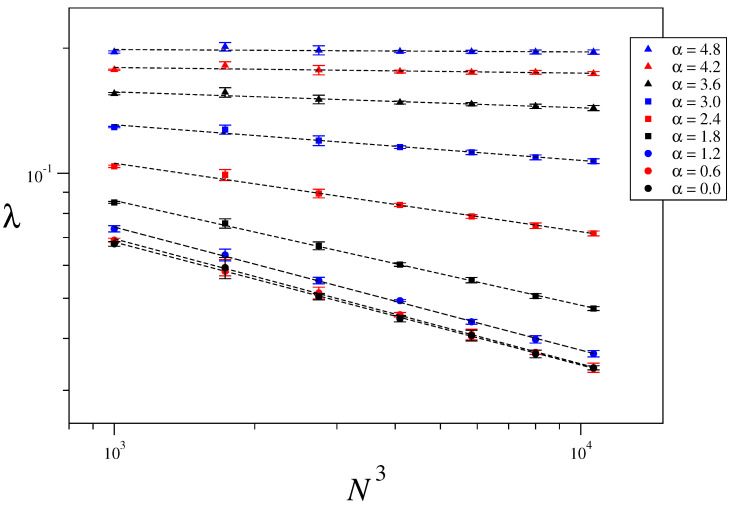
Maximum Lyapunov exponent as a function of the system size for U=5.0, d=3 and different values of α. The considered system sizes are $N={\left(10\right)}^{3},{\left(12\right)}^{3},{\left(14\right)}^{3},{\left(16\right)}^{3},{\left(18\right)}^{3},{\left(20\right)}^{3}$ and ${\left(22\right)}^{3}$.

**Figure 9:**
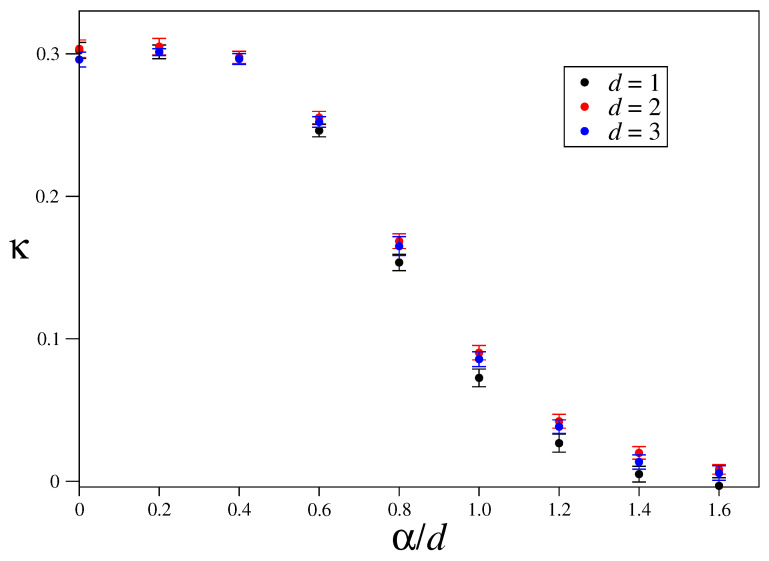
Exponent κ(α/d) in the scaling of the maximum Lyapunov exponent $\lambda ∼N^{-\kappa }$. A collapse of results of data from Figure 6, Figure 7 and Figure 8 is observed.

The maximum Lyapunov exponent characterizes the rate of separation of two initially close orbits. It is defined as $\lambda =\lim_{t\to \infty }\frac{1}{t}\ln\frac{d(t)}{d(0)}$, where d(t) is the distance between orbits, measured in phase space, at time *t*. In order to numerically calculate it, we consider the usual approach given in Ref. [51]. Figure 6 shows the obtained values of the maximum Lyapunov exponents for one-dimensional systems of sizes N=1000,2000,⋯, 10,000, energy per particle U=5.0 and interaction range α=0.0,0.2,⋯,1.6. An average over five different realizations of disorder has been taken in order to improve statistics. In a log-log plot the relation $\lambda ∼N^{-\kappa }$ takes the form of a straight line with slope −κ, which has then been obtained via a least squares fitting. Figure 7 and Figure 8 show the corresponding results for two- and three-dimensional systems for the same values of α/d as in Figure 6 and the system sizes specified in the captions. Figure 9 displays the values of κ obtained in Figure 6, Figure 7 and Figure 8 versus α/d, where a clear collapse of the data is observed, with κ(0)∼0.3 and κ∼0 for $\alpha /d\geq \alpha_{c}\equiv 1.6$. The hypothesis κ=κ(α/d) is thus confirmed. It is worth mentioning that we have an intermediate regime $1\leq \alpha /d\leq \alpha_{c}$ with zero Lyapunov exponent and short-range interactions. This curious anomaly demands further study to check whether it is a finite-size effect or something else.

As mentioned in the beginning of this section, we have used the standard fourth-order Runge-Kutta algorithm for the integration of the equations of motion, by monitoring that the total energy was preserved within a relative precision $10^{-5}$ (at least). However, further simulations using specially designed symplectic methods for Hamiltonian problems [48,49,50] are welcome.

## 4. Conclusions

We have here focused on the classical inertial Heisenberg model in d=1,2,3 dimensions with arbitrarily-ranged interactions, characterized by a power-law decay $r^{-\alpha }$ (α≥0), with the distance *r* between rotators. Such an α-Heisenberg model has been studied previously only in dimension d=1 [47], with a special emphasis on the time evolution of its kinetic temperature, where it was shown to exhibit a clear QSS in the long-range-interaction regime, with its duration decreasing for increasing values of α. Herein, the properties of the model have been preliminary analyzed in d=1,2,3 dimensions, within first-principle numerical calculations, particularly the one-particle moment and energy distributions, as well as the largest Lyapunov exponent.

The Lyapunov exponent scales with the size through the exponent κ, which was shown to depend *only on the ratio*α/d. However, intriguingly enough, the results differ from those already known for the quite analogous α-XY and α-Fermi-Pasta-Ulam models. Indeed, for the XY and Fermi-Pasta-Ulam models, κ monotonically decreases for increasing α/d and vanishes at α/d=1. This fact is in variance with the result obtained for the Heisenberg model, for which κ decreases for increasing α/d but only appears to vanish *above*α/d=1, nearly at α/d≃1.6.

We have illustrated the one-particle momenta and energy distributions in the long-range region with one example, namely for α/d=0.9, and the results have shown a clear collapse of data and were well fitted with a *q*-Gaussian and a *q*-exponential respectively. As found in the recent study of the α-XY model [34], an interesting discrepancy in the values of the index *q* occurred, leading to $q_{L}>q_{E}$. Whether this may be a real effect, or a consequence of finite-size effects, remains a point for further investigation.In addition, we have analyzed similar distributions in the short-range region, namely α/d=1.6. In this case, the results are quite more complex, in the sense that no clear collapse, and consequently, no adequate fitting occurs.

The present numerical analysis is consistent with the theoretical prevision of a change of behavior at α/d=1, in agreement with recent results obtained in other long-range-interaction models, such as a generalized Fermi-Pasta-Ulam model [35,36,37,38,39], complex networks with preferential attachment growth [40,41], and a system of particles under overdamped motion interacting repulsively [42]. In future works we intend to cover the entire α/d≥0 region in order to clarify further this point, and particularly, to investigate why some of the Heisenberg results found herein sensibly differ from those available in the literature for the XY and Fermi-Pasta-Ulam models.
